# Methodologies and Perspectives of Proteomics Applied to Filamentous Fungi: From Sample Preparation to Secretome Analysis

**DOI:** 10.3390/ijms16035803

**Published:** 2015-03-12

**Authors:** Linda Bianco, Gaetano Perrotta

**Affiliations:** UTTRI-GENER Genetics and Genomics for Energy and Environment Laboratory—ENEA TRISAIA Research Center, 75025 Rotondella (Matera), Italy; E-Mail: linda.bianco@enea.it

**Keywords:** fungal proteomics, secretome, filamentous fungi, lignocellulose bioconversion, fungal degradation

## Abstract

Filamentous fungi possess the extraordinary ability to digest complex biomasses and mineralize numerous xenobiotics, as consequence of their aptitude to sensing the environment and regulating their intra and extra cellular proteins, producing drastic changes in proteome and secretome composition. Recent advancement in proteomic technologies offers an exciting opportunity to reveal the fluctuations of fungal proteins and enzymes, responsible for their metabolic adaptation to a large variety of environmental conditions. Here, an overview of the most commonly used proteomic strategies will be provided; this paper will range from sample preparation to gel-free and gel-based proteomics, discussing pros and cons of each mentioned state-of-the-art technique. The main focus will be kept on filamentous fungi. Due to the biotechnological relevance of lignocellulose degrading fungi, special attention will be finally given to their extracellular proteome, or secretome. Secreted proteins and enzymes will be discussed in relation to their involvement in bio-based processes, such as biomass deconstruction and mycoremediation.

## 1. Introduction

The widespread use of fungi in different biotechnological processes can be attributed to their intrinsic characteristics. First of all, they are relatively easygoing organisms and most of them can be grown in fermentors in a quite cheap and easy way. They are eukaryotes, and thus valuable expression hosts for proteins requiring elaborate posttranslational modification. Moreover, they produce a very large array of secondary metabolites, some of which have important activities (*i.e.*, b-lactames). They can secrete an impressive arsenal of extracellular enzymes and proteins, generally referred to as secretome, which represents a powerful biochemical toolkit for the catalysis of a great number of valuable reactions (*i.e.*, regio- and stereo-selective reactions). In the food industry, fungi are used for brewing, baking, winemaking and the production of cheese; in the healthcare industry, they are employed for the production of many pharmaceuticals, such as antibiotics (penicillin, cephalosporins and griseofulvin), immunosuppressants (cyclosporine A and gliotoxin) and anticancer drug (paclitaxel). They are becoming essential to the fine-chemical industry in the production of single-isomer intermediates. With the advent of white technologies, fungi are gaining increasing popularity because of their extremely versatile enzymes as tools to improve biorefinery processes [1]. Indeed, both ascomycete and basidiomycete filamentous fungi have the capacity to secrete large amounts of lignocellulose degrading enzymes that release fermentable sugars from lignocellulosic biomasses, with relevant potential applications in the biofuel industry [2]. Lignocellulose degradation operated by fungi occurs extracellularly, because of the insolubility of cellulose, hemicellulose and lignin. Cellulose and hemicellulose are in fact arranged in a very compact architecture and covered by lignin matrix, forming a physical seal that makes lignocellulose recalcitrant to enzymatic degradation [2].

The fungal ability to efficiently decompose lignocellulose materials arises from a variety of lignocellulose degrading enzymes that can be broadly categorized into two groups. The first group, referred to as the extracellular hydrolytic system, is responsible for polysaccharide hydrolysis into fermentable, monomeric sugars. This system mainly comprises hydrolases, such as cellulases (e.g., glucanases, glucosidases, cellobiohydrolases,) and hemicellulases (xylanases, mannanases, xylosidase), among other enzymes. The second group, termed the ligninolytic system, degrades the lignin moiety of lignocellulose and opens phenyl rings through the use of oxidative ligninases (oxidases, peroxidases, laccases) [1,2]. Different fungi produce unique sets of lignocellulose degrading enzymes, in accordance to their genetic blueprint. Consequently, different fungi may significantly diverge in the way they attack lignocellulose. Indeed, they can use diverse mechanisms distinguishable by the way they make lignocellulose accessible for degradation, by the enzymes involved, and by their behaviour during attack and wood decomposition. These different mechanisms are generally classified as soft-, brown- and white-rot degradations [3]. Soft-rot degradation is widely distributed among both asco- and basidiomycetes and characterized by extensive attack to carbohydrate polymers on wood with low lignin content. Cellulases and hemicellulases are the main enzymes involved in this process, and no ligninases are usually recruited. The filamentous ascomycete *Trichoderma reesei* (*Hypocrea jecorina*) can be considered a model organism for soft-rot degradation; it has been extensively investigated and exploited for the production of cellulases in industrial settings [4]. With very few exceptions, brown- and white-rot degradations are almost entirely a specific trait of basidiomycetes. Brown-rot fungi prevalently depolymerize cellulose and hemicellulose on coniferous wood, without attacking lignin or degrading only a small part of it. An involvement of both hydrolytic and oxidative enzymes have been postulated [3,4]. White-rot degradation is characterized by a massive attack to all components of lignocellulose, lignin included, due to large recruitment of ligninases besides hydrolytic enzymes [3,4]. White-rot basidiomycetes can be further distinguished by their decay patterns. Many of the fungi belonging to this group simultaneously attack lignin, hemicellulose and cellulose whereas some others preferentially work on lignin before attacking carbohydrate moieties [5]. Lignin selective degradation has been proved for *Ceriporiopsis subvermispora* [6], *Phlebia* spp. [7,8], *Physisporinus rivulosus* [9] and *Dichomitus squalens* [8], while other white-rot fungi, such as *Trametes versicolor* [10], *Phanerochaete chrysosporium* [2] and *Irpex lacteus* [11], simultaneously degrade all cell wall components. Selective lignin degraders may have significant biotechnological applications when the removal of lignin is required to obtain intact cellulose such as in biopulping processes and also in procedures where the main objective is to provide an unprotected carbohydrate for subsequent use (e.g., biorefinery) [3,12,13]. However, the molecular mechanisms enabling selective delignification remain unknown.

The extracellular oxidative enzymes involved in lignin depolymerisation include a wide range of oxidases and peroxidases, responsible for the generation of highly reactive and nonspecific free radicals that cleave a variety of carbon–carbon and ether inter-unit bonds. Enzymes such as lignin peroxidase (LiP), manganese peroxidase (MnP), versatile peroxidase (VP) and dye-decolorizing peroxidase (DyP) are considered the most common and representative oxidative enzymes of the ligninolytic system [14]. They act with a mechanism known as “enzymatic combustion” that involves several nonspecific oxidative species; thus, in addition to the main ligninolytic enzymes, a number of accessory proteins (e.g., glyoxal oxidase—GLOX, aryl alcohol oxidase—AAO, pyranose oxidase, methanol oxidase) participate in the process, generating peroxides [14].

Oxidation of complex substrates, such as lignin, is enhanced by low-molecular weight metabolites, termed redox mediators. Examples of these mediators include low-molecular organic acids, fatty acids, phenols, quinones and other aromatic compounds (veratryl alcohol, anisyl and chlorinated anisyl alcohols) produced by fungi [1]. In addition to redox mediators, fungi also produce miscellaneous extracellular polysaccharides (EPs) associated with the mycelia [15]*.* These EPs act as a means of immobilizing extracellular enzymes, attaching the fungus to its solid substrates, providing protection from toxic compounds, preventing dehydration of the mycelium and enhancing carbon storage [16]*.*

Remarkably, the nonspecific nature and extraordinary oxidation potential of the ligninolytic system has also attracted interest in its application in other biotechnological processes, for example, bioremediation of contaminated soils and wastewaters [17].

Fungi showing lignin degrading properties have in fact been proved to degrade and mineralize a large variety of recalcitrant compounds due to their highly reactive enzyme machinery [18]. White-rot fungi utilize the extracellular high-redox-potential heme peroxidases of their ligninolytic system to oxidize an array of environmental pollutants, such as pesticides, organochlorines, polychlorinated biphenyls (PCBs), polycyclic aromatic hydrocarbons (PAHs), synthetic dyes, wood preservatives and synthetic polymers [13].

As already mentioned, the attraction engendered by the extreme flexibility of ligninolytic systems to applications in a large number of fields has led to a drastic increase in the demand for these enzymes in recent times [19]. Thus, the decoding of the genomic blueprint of these organisms is necessary to identify new protein and gene functions, for an efficient design of industrial bioprocesses utilizing these organisms and their enzymes. To date, hundreds of fungal genomes are publicly available. The US Department of Energy (DOE) and Joint Genome Institute (JGI) completed the whole genomic sequence of several filamentous fungi (http://genome.jgi.doe.gov/programs/fungi/index.jsf; http://jgi.doe.gov/our-science/science-programs/fungal-genomics/recent-fungal-genome-releases/) and started, in collaboration with several international research teams, the 1000 Fungal Genome (1KFG) project, to facilitate the sequencing of fungal genomes across the Kingdom Fungi with the objective to significantly advance genome-enabled mycology (http://1000.fungalgenomes.org/). The availability of sequenced genomes and the improvements in mass spectrometry (MS) instrumental resolution enabled and sped up fungal proteome analysis. Nowadays, the major challenge in modern fungal biology is the comprehension of the expression, function and regulation of the entire sets of proteins encoded by fungal genomes. Proteomics is becoming an essential methodology of functional genomics, as it provides information at the protein level, which does not necessarily correlate to mRNA amounts, because of mRNA and protein turnover [20]. Protein information is particularly relevant in eukaryotic systems, such as lignocellulose degrading fungi, because it offers insight into location-specific analysis (subproteome), as well as into post-translational modification, such as phosphorylation, glycosylation and signal peptide, that play a pivotal role in protein activity and localization. Moreover, information at the protein level strongly supports the determination of the relation between a given genome and the corresponding phenotypes.

In this review, we will provide an overview of the proteomics strategies used to study filamentous fungi, from sample preparation to recent advances on MS-based proteomics. Special attention will be given to lignocellulose degrading fungi; representative examples of their secretomes will be discussed in relation to their involvement in bio-based processes, such as biomass deconstruction and bioremediation.

## 2. Sample Preparation for Fungal Proteomics

For all proteomic studies and applications, protein extraction and sample preparation represent the crucial steps for optimal outcome, because they can bias protein yield and affect biological activities, structural integrity of the specific target proteins. Filamentous fungi may contain proteins up to 31% of their total dry weight; nevertheless, they are considered a recalcitrant biological material from which protein extraction is challenging for a number of reasons [21]. Depending on the species, they possess an exceptionally thick and robust cell wall that makes the cell disruption process very challenging [22]. Moreover, they secrete proteases, which can influence extraction procedures, causing protein degradation. Additionally, there are special requirements for the isolation of secreted proteins, due to low protein concentrations and variable amounts of fungal metabolites in liquid culture media. For total protein extraction, an ideal protocol would reproducibly capture all the protein species in a proteome, with low contamination of other molecules. To this aim, different procedures for protein extraction, precipitation or solubilisation have been reported in literature, to circumvent the aforementioned limitations and maximise protein yield.

Mycelia are generally isolated by filtration [23], since centrifugation does not produce a compact pellets, making the subsequent washing steps more difficult. Different cell-breaking systems have been tested in early studies [24,25,26], revealing a greater efficacy of traditional mechanical systems (e.g., mortar grinding) than those based on chemical or enzymatic extraction [27,28,29,30] A variety of extraction buffers have been tested in combination with a grinding procedure. Kim and colleagues adopted Mg/NP-40 extraction buffer to extract total proteins [31]. Successively, this protocol was optimized by replacing NP-40 with CHAPS and named Mg/CHAPS [32]; it gave better results and resolution, when compared with the classical Tris/EDTA [33]. Alternatively, Shimizu and Wariishi developed a sample preparation method based on fungal protoplast, as they possess metabolic activities similar to those seen with intact mycelial cells [34].

Protein precipitation is generally achieved with 10% trichloroacetic acid (TCA) in acetone with 0.07% 2-mercaptoethanol or 20 mM DTT. Although the combination of TCA and acetone is usually more effective than either TCA or acetone alone [32], other mixtures (e.g., 100 mM ammonium acetate in methanol) have been also described for fungi [35]. TCA precipitation protocol is based on protein denaturation under acidic and hydrophobic conditions that minimize protease activities, protein degradation and modification; in addition, it promotes sample concentration, removing interfering compounds that can affect isoelectric focusing (IEF). Despite these undeniable advantages, protein solubilisation after TCA precipitation is problematic. Extensive washing is required to remove residual TCA [23], with consequent loss of samples. To overcome this challenge, a sodium hydroxide-based protocol has been developed by Nandakumar *et al.* [36], to improve solubilisation of TCA-precipitated proteins. Other works reported the use of chaotropes (urea and thiourea) [37], new zwitterionic detergents [38,39], and acidic extraction solution to increase gel electrophoresis resolution, reducing streaking caused by fungal cell wall [40]*.*

Extracellular enzymes of saprophytic fungi such as laccases, peroxidases (lignin, versatile and manganese peroxidases) cellulases and xylanases are of such importance as to define a new research field in proteomics, the so-called fungal secretomics. It has been described as the combination of native proteins and cell machinery involved in their secretion [21]. Saprophytic fungi “can digest their food and then eat it” [41]; therefore, secretome-related studies are particularly relevant in understanding the large number of extracellular enzymes necessary to digest a plethora of potential substrates [42]. Most of the fungi cultivated under laboratory conditions secrete proteins in low concentrations in the growing media, typically 5–50 µg/mL [43]. Moreover, the simultaneous presence of a wide range of interfering compounds, such as polysaccharides, organic and fatty acids, mucilaginous material, phenols and other aromatic compounds, can interfere with the protein quantification via spectrophotometric measurements, resulting in a strong overestimation of the total amount of proteins [43,44,45]. Various strategies to enhance the coverage and detection of secreted proteins have been reported in the literature. Francisco’s group provided pioneering contributions to this field, establishing a sample preparation protocol for fungal secretome, based on the enzymatic or chemical deglycosylation to improve secreted protein analysis by proteomic approaches [45]. Fragner and collaborators [43] found that a considerable amount of interfering gelatinous material can be removed from liquid culture supernatants by high speed centrifugation. The authors tested a combination of different methods, namely precipitation by phenol/methanol, TCA/acetone, chloroform/methanol or ethanol, optimizing a precipitation protocol using TCA/ice-buffered acetone [43]. Some authors reported the use of filtration (0.22 µm) combined to 10% TCA precipitation (at 4 °C) and ice-cold acetone washes, to clarify supernatants and extract secretome [46], while some others described the use of filtration followed by freeze-drying or dialysis in a tangential ultra-filtration system to purify the whole fungal secretome [47,48]. In literature, secretome extraction in native condition has been also reported [49,50], demonstrating that the enzymes secreted into the culture medium interact with each other and generate complexes.

To date, most of the fungal proteomic studies have been focused on mycelial extracts and/or secretome. Nevertheless, many important biological processes occur within specific organelles; thus, “subproteomics”, described as the proteomic analysis of defined organelles [42], is a valuable tool to implement fungal proteomics. The critical steps in fungal subproteomics are detailed by Ferreira de Oliviera *et al.* [51]. Fungi are characterized by a polarized growth supported by microtubules that give rise to the clustering of the different organelles, making their separation more difficult. According to the authors, special attention is to be given to the cell disruption procedure, which should be accomplished in isotonic buffers containing protease inhibitors. French press or mortar grinding are to be preferred to bead milling, unless lysis buffers under extreme conditions (such as boiling or the use of detergents) are used [51]. After debris elimination through filtration or low-speed centrifugation, the authors propose the use of free-flow electrophoresis (FFE) for organelle enrichment [51]. Proteomic analysis of specific cell compartments is an exciting and expanding field that promises to give additional insights into location-dependent processes, for example, biodegradation mechanisms involving the cytochrome P450 monoxygenase system.

## 3. Fungal Proteomics

In proteomic research, proteins are usually identified by the mass-to-charge ratio of their peptides and fragments. Consequently, sample separation prior mass analysis is generally required to reduce sample biological complexity, in order to lower the risk of unambiguous identifications. The major separation technologies commonly used in proteomics can be divided into gel-based and gel-free approaches.

### 3.1. Gel-Based Approaches

Gel-based separation techniques rely on one-dimensional or two-dimensional polyacrylamide gel electrophoresis (SDS-PAGE or 1-DE and 2-DE), which are very popular methods for their robustness and simplicity. For several decades, SDS-PAGE has been one of the most used tools for the separation and pre-fractionation of protein extracts. In fungal proteomics, SDS-PAGE has been used to study hydrophobic proteins, such as membrane and cell wall embedded proteins. As a matter of fact, one of the earliest intracellular proteomic studies in filamentous fungi biology was carried out on white-rot fungi *P. chrysosporium* and *Lentinula edodes* [52]. In this work, the authors studied iron-responsive plasma membrane and cell wall proteins by using SDS-PAGE, due to the impossibility to visualize them by 2-DE. Schmidt *et al.* [53] applied the same approach to inspect mitochondrial outer membrane of *Neurospora crassa*, a filamentous fungus secreting complex cocktails of enzymes that completely depolymerize cellulose and other plant cell wall polysaccharides [53]. Nowadays, SDS-PAGE coupled with MS is becoming a very popular workflow in proteomics; this approach, termed GeLC-MS/MS, is used in different research fields for its effectiveness in the identification of hydrophobic as well as low-molecular-weight proteins, enabling higher proteome coverage [54,55]. In a multidisciplinary approach, GeLC-MS/MS was used to characterize secreted proteins from *Penicillium funiculosum* grown under industrial process fermentation, resulting in the identification of proteins not detectable with other used complementary approaches [56]. Pre-fractionation on SDS-PAGE was also used to inspect the secretome associated with growth and upon carbon source depletion in *Aspergillus niger*, a soil-dwelling filamentous fungus with a high capacity for decomposing plant materials [57]. This study corroborates the *in silico* predictions of extracellular enzymes. The same technical approach was used to investigate protein secretion in *Aspergillus niger*, with particular respect to the protein composition of secretory organelles upon d-Xylose induction [58]. GeLC-MS/MS was also applied to investigate the efficiency of selected secretomes in sugar release from micronized wheat straw [59]. By using this methodology, unexpected potential in lignocellulose degradation was shown for the basidiomycetes *Ustilago maydis*.

A variation of SDS-PAGE, termed Blue Native PAGE (BN-PAGE), has been extensively used to analyze native protein complexes from biological membranes as well as protein–protein interactions in water soluble proteins [60]. In fungal molecular biology, BN-PAGE has been used to investigate the secretome of *Penicillium purpurogenum* and *Trichoderma harzianum* [49,50]. The mentioned studies revealed extracellular enzymes interact with each other to form high molecular weight, catalytically active complexes, whose expression and assembly depend on the used carbon source. Given the biotechnological importance of *Trichoderma* and *Penicillium* sp. as producers of hydrolytic enzymes, this approach provided a valuable insight into the stoichiometry of these complexes.

Despite the simplicity and versatility of SDS-PAGE, 2-DE is currently the dominant platform in gel-based proteomic studies. The first work studying filamentous fungi by 2-DE was carried out by Grinyer and collaborators, in 2004 [25]. By combining 2-DE with matrix-assisted laser desorption/ionisation time-of-flight MS (MALDI-TOF) and liquid chromatography mass spectrometry (LC-MS/MS), they started the first systematic mapping of *Trichoderma harzianum*, a lignocellulolityc filamentous fungus. Hundreds of proteins were mapped on 2-D gels and a total of 25 proteins were successfully identified from the whole-cell protein reference map. On the basis of this established method, further studies mapping intracellular and extracellular fungal proteomes were published, boosted by the accomplishment of numerous fungal genome projects, which increased protein identifications rate. As an example of the progress in fungal proteomics research supported by fungal genome sequencing, here we report the case of *Penicillium chrysogenum* [23,61,62]. After the genome publication, *Penicillium chrysogenum* was subjected to proteomic investigation to study both intracellular and secreted proteins. Mycelia total proteins were separated by 2-DE and a total of 976 spots were resolved and analyzed by peptide mass fingerprinting (PMF) and tandem MS. 848 out of 976 detected spots were successfully identified, representing 950 proteins, because some spots contained more than one protein [61]. *Penicillium chrysogenum* extracellular proteome investigation revealed a total of 317 spots on 2-DE reference map. Out of them, 279 were effectively identified by PMF and tandem MS [62]. Intracellular and secretome reference map were also elaborated for other filamentous fungi of industrial relevance, such as *Phanerochaete chrysosporium* and *Aspergillus niger* [63,64,65].

Although 2-DE has been widely used to generate protein maps, it has been also applied in comparative studies to examine differences among diverse experimental conditions, strains or mutants. For example, the proteome response of *Phanerochaete chrysosporium* to vanillin addition has been evaluated by comparative 2-DE [66], revealing pivotal proteins and enzymes involved in the metabolism of aromatic compounds. Salvachúa *et al.* [48] applied the same method to investigate the proteins secreted over the time by *Irpex lactus*, a basidiomycete white-rot fungus, grown on wheat straw. The temporal alteration of *Irpex lactus* secretome provided an insight into the lignocellulose degradation mechanism [48].

The major concern in 2-DE-based comparative analysis is the error in protein ratio, due to gel-to-gel reproducibility. Even two identical protein samples run on separated 2-DE gels produce similar but not identical protein map. The introduction of DIGE technology (Differentially-In-Gel-Electrophoresis; 2D-DIGE) made a huge step forward in overcoming this limitation. 2D-DIGE relies on the pre-electrophoretic labelling of samples with one of three spectrally resolvable fluorescent CyDyes (Cy2, Cy3, and Cy5), whereby up to three different samples can be simultaneously run on the same 2-DE gel [67]. Inter-gel variability is minimized by the use of an internal standard, co-resolved on the gels and used to normalize protein spot ratios. Recently, dos Santos Castro and co-workers used 2D-DIGE to investigate lignocellulose-degrading enzyme production in *Trichoderma reesei* in response to different carbon sources [68]. Rahmad and collaborators [69] used the same methodological approach to examine the developmental stages of *Termitomyces heimii*, an edible basidiomycete fungus that has a symbiotic relationship with termites. Differentially detected protein spots shed some lights on the poorly understood development process of this fungus, which is also one of the most difficult mushrooms to cultivate.

The introduction of DIGE technology increased the overall sensitivity and resolution power of the gel-based proteomic approaches. The uniqueness in easy visualization of protein isoforms renders 2-DE and 2D-DIGE extremely informative; nevertheless, they suffer from intrinsic limitations. Beside the fact they are still time consuming and laborious (user intervention is very often required in quantitative analysis to manually correct mismatching in image analysis software and improve spot matching accuracy), they have demonstrated poor performance for less common and hydrophobic proteins as well as for proteins with extreme isoelectric points or sizes. Moreover, the co-migration of multiple proteins in a single spot might complicate comparative quantification. In addition, the percentage of proteome coverage reached by these techniques is low, approximately 10% of the total predicted proteins. Regarding this aspect, it should be taken into account that only a fraction of the total fungal genome expresses proteins under specific physiological phases; different sets of proteins might be expressed under different conditions and environments [23].

### 3.2. Gel-Free Approaches

In recent years, intrinsic limitations of gel-based proteomics oriented the scientific community in favor of alternative approaches, globally known as gel-free proteomics. Among different possible approaches to study proteins, MS-based proteomics represents the most attractive technology that should probably be considered as complementary to gel-based applications rather than a mere replacement. In fact, no single method can provide exhaustive information for all the protein components in a complex mixture, and different methodologies usually offer complementary information, which can be integrated into more detailed analyses. MS-based proteomics is characterized by a great variety of approaches and instrumentations. Their detailed description, extensively revised elsewhere [70,71,72], is beyond the scope of this review. Here, we discuss the most common MS-based approaches, among which shotgun proteomics is by far the most widely used. In this methodological approach, complex peptide mixtures are generated after in-solution proteolytic digestion and resolved using different fractionation techniques, before mass analysis. For a deep exploration of complex proteomes, fractionation based on orthogonal physicochemical properties is the method of choice. Multidimensional protein identification technology (MudPIT) developed by Yates’ group [73] combines multidimensional liquid chromatography (SCX-strong cation-exchange chromatography- and RP-reversed-phase chromatography-) to tandem MS to separate and analyze complex peptide mixtures. MudPIT investigation was applied in fungal proteomics to study uredospores germination in *Uromyces appendiculatus*, a phytopathogenic filamentous fungus [74]. Comparative analysis was performed comparing protein accumulation in germinating asexual uredospores to protein accumulated in the inactive spore. Relative protein concentration was estimated by counting the numbers of tandem mass spectra assigned to peptides for each detected protein [74]. MudPIT was also applied to complement expression data of *Neurospora crassa* grown on *Miscanthus* or cellulose-containing media [75], identifying a number of genome-predicted cellulases. This strategy was also used to investigate *Fusarium solani* proteome, confirming the presence of 400 expressed proteins in the culture, among which several lignocellulolytic enzymes [76].

The rapid advancement in MS-technologies is paving the way for the integration of high-throughput processes in proteomics, for faster analyses of larger number of proteins. MudPIT was first applied to characterize the proteome of the single-cell fungus *Saccharomyces cerevisiae* [73]. This investigation yielded a total of 1484 identified proteins, including scarce and rarely seen proteins like transcription factors and protein kinases. The development of new and higher performing MS equipment, characterized by improved scan rates, sensitivity, resolution and mass accuracy, led to progressive improvements of *Saccharomyces cerevisiae* proteome coverage, identifying even larger numbers of proteins, reaching a proteome coverage close to 97% of the genome-predicted proteins [77,78,79]. Nowadays, the majority of *Saccharomyces cerevisiae* proteome can be routinely covered in less than a day with high reproducibility and sensitivity [80]. To the best of our knowledge, deep proteome strategy and technologies have been applied to yeast and mammalian cells [81,82], showing a sharp delay of proteomic research on filamentous fungi when compared to other model systems [79,83,84].

MudPIT-based shotgun approach is a powerful and pivotal technology, with regard to the number of proteins identified when compared to gel-based methods. Nevertheless, for meaningful modelling of biological data, quantitative analysis, at least in relative terms, is required. Gel-free quantitative proteomics can be broadly categorized into label-based and label-free methods. In principle, labelling techniques used in quantitative proteomics do not alter peptide chromatographic and ionisation properties; labelled and unlabelled peptides can be recognized from each other by the mass-shift signature produced by the different isotopic forms. The relative quantification can be achieved by comparing their respective signal intensities [72]. The most popular techniques in quantitative proteomics are: (a) ^18^O chemical labelling (Table 1) [85], where ^18^O is incorporated into peptides during proteolytic digestion, that takes place in the presence of heavy water; (b) ICAT (Isotope-Coded Affinity Tag) (Table 1) [20], where proteins are labelled with specific reagents containing a protein reactive group, a linker with light or heavy isotopes and a biotin purification tag, for peptides recovery after proteolytic digestion; (c) iTRAQ (Isobaric Tags for Relative and Absolute Quantitation) (Table 1) [86], that uses isobaric mass tags to introduce mass-balanced labels, able to generate reporter ions upon peptide fragmentation; (d) TMT (Tandem Mass Tag) (Table 1) [87], that uses isotopomer labels, similar to iTRAQ, and releases “daughter ions” in MS/MS analysis. In the proteomics community, iTRAQ and TMT isobaric reagents are gaining popularity over other methods due to their capability of multiplexing up to eight different biological samples with iTRAQ and 10 samples with TMT.

**Table 1:** The most common isotopic labels.

Labelling Strategy	Reaction Mechanism or Reagent Structure
(a) ^18^O chemical labelling The incorporation of two ^18^O occurs at *C*-terminus of lysine and arginine, during proteolytic digestion.	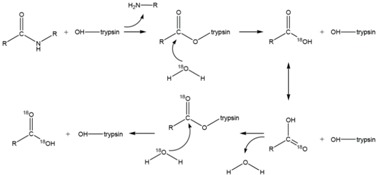
(b) ICAT The ICAT reagent consists of cysteine-directed reactive group, a polyether linker region containing deuteriums (heavy reagent) or hydrogens (light reagent), and a biotin group that allows purification of labeled peptides.	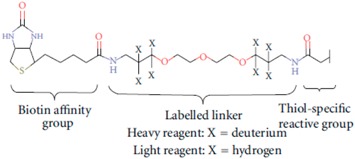
(c) iTRAQ	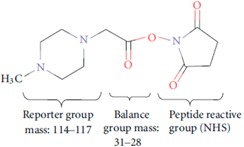
(d) TMT	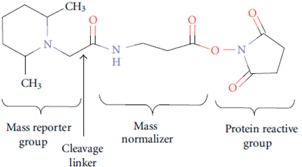

Although a toolbox of different approaches for relative quantitation is currently available, the mentioned techniques have been scarcely implemented in fungal proteomic research, with the exception of iTRAQ. One of the earliest works applying label-based quantitative proteomics was carried out by Taylor *et al.* [29]. By using iTRAQ, the authors investigated protein accumulation in the plant pathogenic filamentous fungus *Fusarium graminearum*, in response to mycotoxin stimulation. One hundred and thirty out of 435 identified proteins showed significant changes, revealing their involvement on the onset of mycotoxin synthesis. iTRAQ was also used to investigate the protein expression changes in *Aspergillus fumigatus*, a pathogenic filamentous fungus, to profile its proteome in response to the antifungal drug, caspofungin [88,89]. *Aspergillus fumigatus* secretome was further inspected by Liu and coworkers [90]. The fungus was grown on different carbon sources and, through the iTRAQ approach, a strong enzymatic toolbox for lignocellulose biomass deconstruction was revealed. By using a similar technical approach, secretome alteration of industrially relevant lignocellulose degrading fungi, such as *Aspergillus niger* wild type and mutant strains [46], *Phanerochaete chrysosporium* [91,92] and *Trichoderma reesei* [47,93], were dug into deeply, detecting a number of biotechnologically significant enzymes. Finally, a novel isobaric tag, named deuterium ((2)H) isobaric amine-reactive tag (DiART) [94], has been introduced for quantitative proteomics and used to study *Aspergillus nidulans* cell wall proteins [95].

The label-based quantification strategies here mentioned rely on chemical labelling. This type of approach is quite expensive, thus preventing large-scale experiments. Moreover, it suffers from intrinsic limitations, due to sample variability and technical biases which are introduced when labelling occurs after protein extraction or proteolytic digestion. A valuable alternative approach is represented by metabolic labelling, where protein labelling takes place during protein synthesis, because of the addition of stable isotopes into the growth medium. Quantification can be achieved by comparing MS spectra of “heavy” and “light” forms. The most popular strategies belonging to this group are SILAC (stable isotope labelling by amino acid) and ^14^N/^15^N labelling [96,97]. SILAC exploits the *in vivo* incorporation of light and heavy amino acids, typically lysine and arginine (labelled with ^13^C and/or ^14^N). In ^14^N/^15^N labelling, stable isotopes are introduced to the whole cell or organism through the growth medium, containing ^15^N-labelled inorganic salts (usually K^15^NO_3_). In fungal proteomic, SILAC has been extensively applied to *Saccharomyces cerevisiae* [98]. To the best of our knowledge, there are very few works applying SILAC or ^14^N/^15^N labelling to filamentous fungi, despite their great potential in fungal proteomic research. One of these studies was carried out by Phillips *et al.* [99] and directed to investigate the secretome of *Neurospora crassa*, cultivated on cellulose. By applying a combination of methods and a slightly modified version of SILAC, the authors achieved the absolute quantitation of four proteins of interest, expected to be the core of fungal cellulose degradation.

Nowadays, there is a growing interest for label-free quantitation methods as an attractive alternative to both chemical and metabolic labelling methodologies. Label-free approaches are relatively easy to perform, inexpensive, unaffected by labelling process-related bias. Moreover, they can be applied to any kind of biological sample and unlimited numbers of samples can be compared. In a typical label-free quantitative proteomic experiment, samples are individually analyzed and compared after independent analyses, typically using spectral counting or peak intensities measurement, which approximate protein abundance [100]. Spectral counting is becoming very popular in proteomic research, due to its simple procedure based on the rationale that the more abundant the protein is, the more peptides can be identified from it [73]. The number of tandem mass spectra recorded for a peptide in a sample linearly correlates with its molar amount [101]; therefore, the sum of spectral counts for peptides associated with a particular protein can be used to estimate protein amount [102,103]. Because larger proteins generate more peptides and consequently more MS/MS events, spectral counting approaches must take into account protein attributes (e.g., length, molecular weight, absolute and relative amino acid frequencies, observable peptides, *etc.*), as implemented in several spectral counting methods, such as APEX (Absolute Protein Expression) [104], NSAF (Normalized Spectra Abundance Factor) [103], and emPAI (Exponentially Modified Protein Abundance Index) [105]. Cooper *et al.* [74] used spectral count NSAF-based method to achieve relative protein quantification in *Uromyces appendiculatus* germinating and inactive spores. For each protein, they summed up the spectral counts for all identified peptides. The resulting sum has been normalized dividing it by the protein mass, and further, by the sum of all adjusted spectral counts for all identified proteins [74,103]. Through this approach, the authors revealed a number of differentially accumulated proteins in germlings. Adav and collaborators applied label-free emPAI-based quantitative proteomics to investigate the lignocellulolytic enzyme secretion profile of *Thermobifida fusca*, a thermostable filamentous fungus, with relation to different lignocellulolytic biomasses [106]. emPAI method has been implemented into Mascot platform (Mascot, Matrix Science), based on the following equations:

(1) $$\text{PAI}=\frac{Nobsd}{Nobsdl}$$

emPAI = 10^PAI^ − 1 (2)

where Nobsd is the number of experimentally observed peptides per protein and Nobsdl is the number of theoretically observable peptides *per* protein [106,107]. emPAI approach applied to *Thermobifida fusca* was able to detect a number of cellulose, hemicellulose and lignin degrading proteins differently accumulated, depending on different lignocellulose biomasses used for solid state fermentation [106].

Label-free quantitative proteomic is far from being fully exploited in fungal proteomic research, as revealed by the limited number of papers published in this context. Nevertheless, it is worth noting an interesting aspect of this approach, whereby new proteins can be discovered from old spectral datasets, when new information is added to the reference databases or when new sets of samples become available. This is possible because samples are independently analyzed and quantified after MS analysis; thus, the obtained data is not fixed and can be used in other contexts as well, provided that LC-MS/MS experiments are conducted under the same conditions and experimental steps are kept to a minimum to control reproducibility. Each of the methods here mentioned has its own merits and drawbacks; the choice of the optimal method should be evaluated in accordance with the experimental design and downstream needs.

## 4. Secretome for Targeted Bioprocesses: From Efficient Biomass Deconstruction to Mycoremediation

The extracellular proteome, globally referred to as secretome, is defined as the totality of the proteins secreted from a cell or an organism at a given time, under a certain condition. It includes proteins released into the surrounding medium, proteins attached to the outer cell wall as well as proteins involved in the secretory pathway [108]. Due to the advancement of fungal genome projects, secretomes can be *in silico* predicted (bioinfosecretomes), based on the identification of secretion signals (SP, signal peptide) in the putative proteins corresponding to the annotated genes [109]. However, unconventional protein secretion pathways are ER/Golgi independent and do not require the presence of SP in the sequence to be exported [109,110]. Moreover, actual secretomes can significantly diverge from bioinfosecretome, as they are transcriptionally regulated in response to an array of environmental conditions, including temperature, pH, carbon sources. Thus, environmental conditions can severely affect protein expression and accumulation trends, producing dramatic changes in the extracellular protein pattern. The composition of real secretomes is further complicated by the fact protein expression is species-specific and substrate-dependent. Analyses of wet secretomes are necessary to provide more detailed information about the set of proteins released under specific growing conditions and/or at a given time; proteomics offer valuable analytical tools for this purpose.

**Figure 1:**
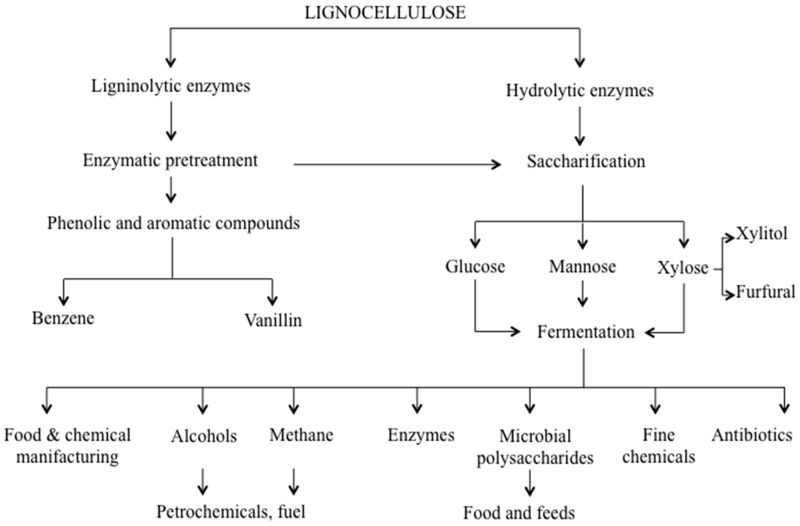
Lignocellulose bioconversion steps taken by fungal lignocellulose degrading enzymes.

Fungi can adapt to different environments and adjust their metabolism to accommodate detrimental conditions, by modulating the dynamic nature of secretome and degrading complex substrates outside the cell before their intake. In this regard, it has been written that “fungi digest their food and then eat it”, differing from animals that first “eat their food and then digest it” [41]. Indeed, fungi have the ability to extracellularly decompose a large variety of substrates, lignocellulose included (Figure 1).

Lignocellulose is the most abundant, virtually inexhaustible, renewable carbon resource. Lignocellulose materials having different nature, origins and compositions (woody, non-woody crops, industrial and municipal residues) can differently stimulate and elicit fungal secretomes. As already mentioned, fungi can decompose lignocellulose using a combination of a great variety of degrading enzymes. Such enzymes have been shown to be promising candidates for several biotechnological applications [1,3,12,13]; consequently, they have attracted the attention of modern biotech-oriented companies. The progress of molecular biology and the growing interest towards lignocelluloses as a renewable carbon resource are in fact responsible for the huge evolution of white biotechnologies, where fungi and their enzymes are useful tools to generate industrially relevant products, such as biofuels, value added compounds and chemicals (Figure 1) [1]. From this perspective, exploration of fungal biodiversity through their secretome is currently a valuable method to detect new strains or stronger performing enzymes which can break down lignocellulose much more efficiently, withstanding pH and temperature inhibitory effects [109,111,112]. In this framework, it is worth mentioning that *Trichoderma reesei*, whose secretome has been extensively studied [68,111,112,113]. Despite the relatively small number of cellulases-encoding genes for lignocellulose-degrading fungus [114], *Trichoderma reesei* is an extraordinarily efficient producer of cellulases, commonly used for biomass hydrolysis in industrial settings [3,4]. Mutants of *Trichoderma reesei* have been widely investigated in different culture media under various conditions, resulting in the selection of industrial strains secreting extracellular cellulases with up to 100 gL of culture [115].

In parallel to strain selection, analyses of fungal secretomes were also focused on the detection of more efficient enzymes, with higher resistance to pH and temperature variations. pH effects were evaluated on *Aspergillus niger*, revealing a number of hydrolytic enzymes able to completely hydrolyze cellulose and hemicellulose at extended pH conditions [46]. Instead, temperature effects were evaluated on *Aspergillus fumigatus*, identifying new thermostable glycosyl hydrolase proteins [116]. The discovery of new thermostable enzymes is an aspect of pivotal importance to improve the overall efficiency of lignocellulose hydrolysis; in fact, enzyme stability at high temperature is one of the major limits to enzyme applications at the industrial level. Given the complexity of lignocellulose and the impressive variability of fungal extracellular proteins, proteomic research has more recently shifted toward the characterization of enzyme mixtures, in order to support the development and optimization of specific enzymatic cocktails for a more efficient hydrolysis of biomasses. Particularly relevant is the composition of cocktail preparations. Chundawat *et al.* [117] have demonstrated that in several commercial preparations the major enzyme was Cel7A, followed by Cel6A; endoglucanases and hemicellulases accounted for the 10% of total proteins; interestingly accessory proteins, such as swollenin and cip proteins, were present in considerable amounts, up to 4% and 3%, respectively. The role of these accessory proteins in saccharification is not yet fully understood.

Although it is generally accepted that the classic mechanism by which aerobic fungi depolymerize lignocellulose is driven by the synergism among free hydrolytic enzymes, some works have shown that fungal secretomes may contain the major hydrolytic enzymes assembled into multi-enzymatic complexes. The formation of multi-enzymatic complexes has been reported in the thermophilic fungus *Chaetomium* spp*.* [118], and more recently for *Trichoderma harzianum* and *Penicillium purpurogenum* [49,50,119]. For the latter, secretome analysis carried out in native conditions led to the identification of different subunits, including glucan 1,4-α-glucosidase, β-1,6-glucanase, α-l-arabinofuranosidase, alcohol dehydrogenase, endo-1,4-β-xylanase, cell wall protein PhiA acetylxylan esterase 2 [49,119]. However, how proteins associate with these complexes is far from being fully revealed. This is an important point to be addressed in order to achieve the design of stronger performing enzyme cocktails. In fact, specific enzyme activities could be affected by protein-protein interactions, and these aspects should be taken into account for the optimization process.

To design an efficient degrading enzyme cocktail, protein amounts and their optimal relative ratios also need to be considered. Thus, several attempts to quantify lignocellulolytic protein expression during biomass degradation were also reported in the literature [109,112]. Remarkably, most of these quali-quantitative studies were carried out on white-rot fungi. As previously described, white-rot basidiomycetes developed the distinctive ability to decompose and mineralize lignin. They attack lignin using the main ligninolytic enzymes, represented by MnP, VP, LP. Besides them, a number of accessory enzymes participating in the process have been recognized, including hemeperoxidase, chloroperoxidases, dye decolorizing peroxidases, in addition to glyoxal oxidases, aryl alcohol oxidases, pyranose dehydrogenases, and methanol oxidases. It is worth noting that not all white-rot basidiomycete secretomes expressed all of these enzymes; some of them are released only under specific conditions [109]. Beside their main involvement in lignin degradation, ligninolytic enzymes seem to play a role also in cellulose and hemicellulose breakdown. The synergism between cellulases and oxidative enzymes was already reported in literature [120]. Consequently, a double interest in lignin degrading enzymes is nowadays emerging; first, they need to be investigated as accessory proteins to improve the overall performance of enzyme cocktails. Secondly, they need to be investigated for their role in biological pretreatment of lignocellulosic biomasses to remove recalcitrant lignin, enhancing saccharification yields. Classical biomass pretreatment, usually accomplished by expensive and energy consuming physico-chemical processes [121,122], generate various interfering compounds (e.g., organic acid, furfurals and phenolic compounds) affecting downstream microbial activities [123].

The white-rot basidiomycete *Phanerochaete chrysosporium* emerged as the model system for studying such ligninolytic enzymes. *Phanerochaete chrysosporium* genome was sequenced in 2004 [124]. Based on computational analysis of more than 10,000 coding sequences, a total of 769 proteins were *in silico* predicted in its bioinfosecretome [125,126]. *Phanerochaete chrysosporium* wet secretome was investigated under a variety of culture conditions [64,92,112,127]. Manavalan *et al.* [91] raised *Phanerochaete chrysosporium* in submerged cultures supplemented with synthetic lignin, cellulose or a mixture of the two. Interestingly, iTRAQ analysis detected no LiPs and MnPs in the secretome stimulated by synthetic lignin, suggesting that the latter might not act as their inductor. On the contrary, enzymes such as copper radical oxidase, isoamyl oxidase, glutathione *S*-transferase, thioredoxin peroxidase, quinone oxidoreductase, aryl alcohol oxidase, pyranose 2-oxidase, aldehyde dehydrogenase, and alcohol dehydrogenase were significantly regulated depending on the substrates [91]. *Phanerochaete chrysosporium* was also cultivated on more complex substrates, such as corn stover, hay, sawdust, sugarcane bagasse, wheat bran and wood chips [92]. Secretome analysis revealed a total of 329 identified proteins, where ligninolytic enzymes accounted for 10% of the total number, whereas hydrolytic enzymes and proteases represented the most abundant groups, accounting for 52% and 20%, respectively. In accordance with the aforementioned results, no LiPs and MnPs were detected in this work. *Phanerochaete chrysosporium* is a very efficient lignin degrader; however, the absence of lignin and manganese peroxidases under the mentioned substrates raises the speculation that lignin degradation can be accomplished through diverse mechanisms. In particular, the identification of several oxidases (e.g., cellobiose dehydrogenase, copper radical oxidase, cellobiose dehydrogenase, glucose oxidase, isoamyl alcohol oxidase, peroxiredoxins, pyranose 2-oxidase, quinone oxidoreductase, iron-containing alcohol dehydrogenase) indicates a putative involvement of such proteins in wood delignification [128,129].

*Phanerochaete chrysosporium* was also used as reference to investigate the secretome of other well-established white-rot fungi, such as *Irpex lacteus* and *Pleurotus ostreatus* [48]. The three species grown on wheat straw produced very different degradation patterns. *Phanerochaete chrysosporium* secreted a plethora of cellulose and hemicellulose degrading enzymes, suggesting a preferential consumption of carbohydrates over lignin. *Pleurotus ostreatus* released more oxidoreductases, probably related to the selectivity towards lignin degradation (ref 3irpex lactus comparison). *Irpex lacteus* produced a similar amount of cellulose and hemicellulose degrading enzymes, but a lower percentage of oxidoreductases, when compared to *Phanerochaete chrysosporium* [48]. Interestingly, the oxidoreductase enzymatic toolboxes displayed by these three fungi significantly differ from each other. *Irpex lacteus* released DyPs, MnPs, cellobiose dehydrogenase, and glyoxal oxidases; *Phanerochaete chrysosporium* released the same enzymes, excluding DyP, but also some others such as LiPs and pyranose 2-oxidase. In contrast, *P. ostreatus* secretome contained mostly VP, MnP, laccases, and glyoxal oxidases [48]. Differences in temporal protein expression profile among fungal strains were also revealed [48]. These differences can be related to the existence of two distinct patterns for fungal biomass delignification, classified as simultaneous and selective delignification [2,6,11], whose mechanisms still remain unknown.

In the attempt to shed light on the unknown mechanism of lignin selective removal, the secretome of *Ceriporiopsis subvermispora* cultivated in media supplemented with complex lignocellulose substrate was investigated at different time points. The sequential enzyme secretion revealed an interesting accumulation of manganese peroxidase and alcohol aryl oxidase at early stages of cultivation [130]. Beside, lignin- and versatile peroxidases were not detected at any point. Significant changes in enzymes related to cellulose and hemicellulose degradation were also found [130].

The integration of secretome data into the formulation of enzyme cocktails for more efficient lignocellulose pretreatment and/or hydrolysis remains nowadays the major challenge; likely, in the near future, efficient enzyme cocktails will be generated on-demand, based on the specific biomass characteristics. A contribution to this regard is given by the recent advancement in meta-omics sciences, particularly meta-proteomics. In nature, various microorganisms producing lignocellulolytic enzymes work synergistically to decompose plant biomasses. Microbial consortia have proved to be attractive sources for exploring novel degrading organisms and enzymes. Meta-proteomics of natural habitats can provide relevant information about the synergism between microbes and their enzymatic arsenal [131,132]. To this purpose, Adav *et al.* [133] co-cultured potent lignin degrading basidiomycetes and effective cellulolytic ascomycotes fungal strains on saw dust. By using such a fashion-like meta-proteomic approach, the authors improved the lignocellulose degradation, highlighting novel enzymes and shedding new lights on synergism and co-operative mechanism. They analyzed single and co-cultured secretomes, revealing that co-culturing stimulated the production of cellulolytic and hemicellulolytic proteins. Different lignin degrading proteins were also expressed in individual and co-cultured secretomes. Data collected suggested that co-culturing stimulated the secretion of glyoxal oxidase, FAD binding domain protein, copper-zinc superoxide and glyoxalase, among ligninolytic enzymes [133].

Beside their role in lignocellulose degradation and their industrial relevance as a source of hydrolytic and ligninolytic enzymes, lignin degrading fungi have been proven to degrade and mineralize a large variety of recalcitrant compounds due to the non-specificity of their enzyme machinery [134]. They have been shown to degrade pesticides, organochlorines, polychlorinated biphenyls (PCBs), polycyclic aromatic hydrocarbons (PAHs), synthetic dyes and polymers [18]. Widespread investigations have been carried out since the mid-1980s to explore their bioremediation capacities [134,135,136]. Nevertheless, no extensive high-throughput proteomic screening has been performed to characterize the fungal enzymatic toolbox in relation to bioremediation potential. Current proteomic technologies provide valuable support in identifying novel strains and new enzymes to be used in fungal biological remediation (mycobioremediation). As shown and discussed in detail above, fungal secretome is highly regulated by environmental stimuli and substrates. The evaluation of secretome profile in response to specific pollutants might provide detailed insights into mycoremediation capabilities. Several works suggest that wood degrading fungi can metabolize pollutants due to their oxidative lignin degrading enzymes. The wood degrading *Phanerochaete chrysosporium* was investigated to elucidate the proteomic response to exogenous vanillin addition [66]. Vanillin is a key intermediate generated during lignin biodegradation that can be also assimilated to a model compound to study aromatic degradation. Interestingly, the authors revealed an up-regulation of homogentisate 1,2-dioxygenase, 1,4-benzoquinone reductases, aldehyde dehydrogenase, and aryl-alcohol dehydrogenase proteins. In parallel, the expression of MnP was activated by the addition of vanillin. In addition, a metabolic switch from the glyoxylate cycle to the tricarboxylic acid cycle was observed [66].

White-rot fungi were also investigated in relation to their potential in wastewater treatment [135]. Olive oil mill wastewater and dry residues are a major environmental problem for their high organic load and antimicrobial properties, especially for Mediterranean countries where most of the world’s olive oil production takes place. Fungal oxidoreductases and hydrolases are promising tools for wastewater bio-treatment. The secretome of the white-rot *Bjerkandera adusta* has been investigated in this context [137]. Distinct changes in the protein composition of oxidoreductases were found in the secretome of this fungus, grown in the presence of dry olive mill residues. A number of class-II peroxidases and aryl alcohol oxidases were more abundantly expressed in comparison to the control sample. Two short manganese peroxidases (MnP1 and MnP6) and one versatile peroxidase (VP1) represented 29% of the relative abundance (NSAF) of detected proteins [137]. *Bjerkandera adusta* is a well-known filamentous fungus involved in recalcitrant dye decoloration [138]. The use of this fungus in the treatment of wastewater produced by textile industries has also been suggested [139]. In our lab, the secretome profile of *Bjerkandera adusta* in response to recalcitrant anthraquinone dyes (Remazol Brilliant Blue R) has been investigated by DIGE technology revealing an interesting accumulation of specific ligninolytic peroxidases [131]. However, the exact role played by these enzymes in wastewater treatment remains unclear.

In the near future, we expect that cutting-edge high-throughput proteomics will be extensively applied to unravel the secretome of filamentous fungi, with particular respect to the less investigated white-rot basidiomycetes.

## 5. Concluding Remarks

Omics sciences, and proteomics in particular, appear to be an outstanding way to decipher the enzymatic toolbox of lignocellulose degrading fungi. Remarkably, these organisms modulate their proteome to accommodate their saprophytic lifestyle. Thus, they can secrete a large number of species-specific extracellular enzymes, whose expression is strongly influenced by environmental stimuli and carbon sources. The proteomics of lignocellulose degrading fungi offers remarkable insights into their mechanisms of action, prospecting new organisms and novel enzymes. The application of cutting-edge high-resolution proteomic techniques will likely boost this process, providing more thorough analyses and unprecedented detail. Deep proteome strategies are expected to shed new light on the less abundant proteins, usually left to the side in favor of the most abundant cellulases, hemicellulases and ligninolytic enzymes. A more efficient exploitation of the potential of lignocellulose degrading fungi will derive from the exploration of their natural habits, by using complementary meta-omic sciences. The meta-investigation of such habits is expected to offer an extensive overview of cooperative organisms and synergistically acting enzymes, thus leading to a more efficient design of bio-based processes. Compared to other meta-omic sciences, metaproteomics is still in its infancy, as it is laborious and technically demanding; however, the progress in DNA sequencing as well as technical advancement in MS are expected to support its extensive application in even more complex decomposing environments.
